# Deqi Sensation in Different Kinds of Acupuncture 

**DOI:** 10.1155/2014/121573

**Published:** 2014-02-09

**Authors:** Cun-Zhi Liu, Gerhard Litscher, Fan-Rong Liang, Jian Kong, Lin-Peng Wang, Lu Wang

**Affiliations:** ^1^Acupuncture and Moxibustion Department, Beijing Hospital of Traditional Chinese Medicine Affiliated to Capital Medical University, 23 Meishuguanhou Street, Dongcheng, Beijing 100010, China; ^2^Stronach Research Unit for Complementary and Integrative Laser Medicine, Research Unit of Biomedical Engineering in Anesthesia and Intensive Care Medicine, TCM Research Center Graz, Medical University of Graz, Auenbruggerplatz 29, 8036 Graz, Austria; ^3^College of Acupuncture and Massage, Chengdu University of Traditional Chinese Medicine, Chengdu, Sichuan 610075, China; ^4^Department of Psychiatry, Massachusetts General Hospital (MGH), Harvard Medical School, Charlestown, Boston, MA 02129, USA; ^5^School of Traditional Chinese Medicine, Capital Medical University, 10 Xitoutiao, Youanmen, Beijing 100069, China

The current issue is the 2013 issue which includes 21 interesting papers.

Acupuncture stimulation elicits Deqi, a composite of unique sensations that is essential for clinical efficacy according to traditional Chinese medicine. In recent years, clinical trials of acupuncture have paid increasing attention to the evocation of Deqi. The physiological mechanism that produces the effect of Deqi has also been explored in several studies but is not well understood.

As mentioned above, this special issue contains 21 papers, of which two papers are related to the characterization of the Deqi during acupuncture treatment. Deqi sensation often occurs during acupuncture treatment and is believed to be important for a successful acupuncture therapy. So far there exist no questionnaires for children. E. Anders et al. created a sentence based questionnaire for children on the basis of the Southampton Needle Sensation questionnaire (SNSQ). Three papers study the interaction between Deqi and acupuncture by neuroimaging technology. They provide evidence to understand neural mechanism underlying acupuncture. Four papers are related to the physiological mechanism of Deqi. These papers describe current knowledge in understanding of Deqi from a physiological aspect. Three reviews are related to the recent advances in Deqi and acupuncture effects. The current evidence base is not solid enough to draw any conclusion regarding the predicative value of natural Deqi for clinical efficacy or the therapeutic value of manipulation-facilitated Deqi. Six papers focus on Deqi in manual acupuncture compared with other types of acupuncture, of which 4 papers introduce the influence of the Deqi sensation by suspended moxibustion stimulation. Two papers adopt randomized controlled clinical trial and multicenter prospective cohort design to compare the clinical effectiveness of Deqi sensation.

Deqi should be taken into account in clinical trials, and more researches are required to understand the underlying mechanisms, as described in this special issue.

*Cun-Zhi Liu* *Cun-Zhi Liu* *Gerhard Litscher* *Gerhard Litscher* *Fan-Rong Liang* *Fan-Rong Liang* *Jian Kong* *Jian Kong* *Lin-Peng Wang* *Lin-Peng Wang* *Lu Wang* *Lu Wang*

